# Coupling coordination and interactive effects of new urbanization efficiency and eco-efficiency—A case study of Fujian Province

**DOI:** 10.1371/journal.pone.0292921

**Published:** 2024-02-08

**Authors:** Yan Cao, Jianchong Wei

**Affiliations:** 1 College of Finance, Fujian Jiangxia University, Fuzhou, China; 2 College of Electronics and Information Science, Fujian Jiangxia University, Fuzhou, China; Qufu Normal University, CHINA

## Abstract

**(1) Background:**

This study explores the coupling and coordinated development of new urbanization and eco-efficiency and their interaction mechanisms from the perspective of efficiency, and it has significant implications for promoting high-quality development and surpassing in China’s regional development.

**(2) Objective:**

The study aims to investigate the spatiotemporal distribution pattern of new urbanization efficiency and eco-efficiency and its coupling and coordinated development relationship.

**(3) Methods:**

Using panel data from 2010 to 2020 for nine cities in Fujian Province, this study employs the undesired super-efficiency SBM model to measure the efficiency of new urbanization and eco-efficiency. Additionally, a spatial panel Durbin model is constructed to analyze the interaction effect between the two efficiencies.

**(4) Results:**

During the study period, both new urbanization efficiency and eco-efficiency in Fujian Province showed a fluctuating upward trend, with higher efficiency in the southeast than the northwest, exhibiting significant spatial agglomeration effects. Despite high double efficiency coupling, coordinated development was low, evolving from near-disorder to barely coordinated. The high coupling and coordination areas were mainly concentrated in the southeast, with gaps between different regions gradually narrowing. There was positive interaction between dual efficiency in the same region, with new urbanization efficiency showing a positive spatial spillover effect on eco-efficiency. Conversely, the spatial spillover effect of eco-efficiency on new urbanization efficiency was not significant.

## 1. Introduction

The new urbanization is a new force that drives China’s economic and social development. It pays more attention to the quality of development while maintaining a focus on the rate of growth, and it emphasizes holistic human development so as to balance development between urban and rural areas, which is the fundamental path and strong support for achieving sustainable and healthy development and shared prosperity in the region [1]. With the increasing demand for resources caused by population agglomeration, industrial agglomeration, and rapid economic development, urbanization has also imposed significant pressure on the ecosystem and environmental carrying function, including ecological environment damage, excessive consumption of resources and energy, unreasonable construction of the land structure, and gradual depletion of land resources, etc. The deterioration of the ecological environment will hinder the urbanization movement, producing a dynamic interaction of mutual constraint and coercion between the two [2]. Under the current new development pattern, in which the domestic big cycle is the main body and the domestic and international double cycles mutually reinforce one another, high-quality development is the overarching theme in all fields of development in China, and saving, intensive, green, and low-carbon development have become essential requirements. According to the report of the 20th National Congress of the CPC, fostering green and low-carbon economic and social growth is the key to achieving superior development. Hence, in the process of urbanization, how to spend the fewest resources to gain the greatest economic, social, and ecological benefits has become the focal point of new urbanization research [3] and is attracting the interest of an increasing number of researchers. However, eco-efficiency is a comprehensive indicator that can simultaneously measure the economy and ecological environment, reflecting the impact of economic output on resources and environment, and is a crucial basis for studying the contradiction between ecological civilization construction and social and economic development [4]. The coordinated development of modern urbanism construction and eco-efficiency has significant strategic importance for supporting comprehensively high-quality regional development in China in accordance with the ecological civilization concept “lucid lakes and verdant mountains are priceless assets.”

Today, urbanization efficiency research is receiving a growing amount of attention. From the perspective of the research area in China, the provinces, counties, the Yangtze River Economic Belt [5], the Yellow River Delta [6], and the major urban agglomerations in the Pearl River Delta, the Yangtze River Delta, Chengdu and Chongqing [7, 8] serve as the research scale, while provincial and municipal research is relatively limited. From the perspective of research content, it focuses primarily on the study of the distribution pattern of urbanization efficiency and its influencing factors, as well as its relationship with industrial agglomeration [9], the influence of industrial structure and environmental regulation [10, 11], and the coupling coordination relationship with economic growth [12]. In terms of research methods, the majority of studies have employed input-output indicator systems and stochastic frontier analysis techniques, including the traditional Data Envelopment Analysis (DEA) model and the Stochastic Frontier Approach (SFA) model, to measure efficiency. However, undesired output issues such as environmental pollution and carbon emissions in the process of urbanization are often ignored, and the ordering of decision-making units with an efficiency of 1 is not considered, so the efficiency of effective decision-making units cannot be fully compared. Even more research has been conducted on eco-efficiency, with a greater variety of studies, more complete perspectives, and more extensive research material. It has been improved to assess industrial eco-efficiency and to analyze its influencing elements for agriculture, industry, tourism, and logistics [4, 13–15]. Currently, the discussion on the interaction between urbanization process and ecological environment has become a research hotspot at home and abroad [16, 17]. Grossman and Krueger [18] were the first to note that the ecological environment curve exhibited an inverted “U” form as the urban economic level increased; afterwards, several researchers confirmed this finding. Yet, when Chinese researchers examined the relationship between urbanization and ecological environment, they discovered that it was S-shaped, U-shaped, and semi-U-shaped [5, 19]. Other research also indicated that urbanization would result in oppression and severe environmental effects [20]. When the conflict between urbanization and ecological environment become more pronounced, the academic community begins to investigate the evolution of their linkage coordination. Yet, the following problems remain across all the existing studies: First, the majority of existing research focuses on constructing an evaluation index system for the two systems of urbanization and ecological environment, and then calculating the coupling coordinating relationship between them based on their complete score index. Few literatures examine the coupling coordinating link between the new urbanization’s efficiency and eco-efficiency, as well as its spatio-temporal change features, from the standpoint of efficiency. Urbanization efficiency often refers to the utilization efficiency of several urbanization input components [21]. Compared with the comprehensive level of urbanization, it can better reflect the quality and efficiency of urbanization construction. Eco-efficiency emphasizes the measurement of the extent of damage to resources and the environment under specific economic output conditions, which is a good indicator of the ecological environment and economically friendly development. Second, most of the studies addressing the relationship between the new urbanization and the ecological environment is one-way, that is, it only considers the impact of the new urbanization on the ecological environment, while ignoring the feedback effect of the ecological environment on the construction of the new urbanization. Furthermore, the majority of the research on the interaction between the two is qualitative, whereas the research on the quantitative analysis is quantitative. Third, in the new urbanization efficiency index system, most studies only focus on social and economic benefits, while ignoring the positive and negative outputs of the ecological environment, and the interpretation of the connotation of new urbanization is not perfect.

By the end of 2021, the urbanization rate of China’s permanent population was 64.72%, and the urbanization rate of Fujian’s permanent population was 69.7%, ranking the top in the country. In addition, Fujian Province, as the first batch of national ecological civilization demonstration areas, has been committed to the development of green economy and low-carbon circular economy for a considerable amount of time, resulting in relatively high ecological environment indicators. Thus, the focus of this work is on nine prefecture-level cities in Fujian Province. It develops two input-output evaluation index systems to evaluate the efficiency of new urbanization and eco-efficiency, respectively. The undesirable output super-efficiency SBM model is then utilized to determine the dual efficiency values of these cities from 2010 to 2020. The study examines the spatiotemporal patterns of the dual efficiency rate and the relationship between its coupling and coordinated development. In addition, a spatial Dubin model (SDM) is created to study the mechanism of interaction between the two measurements. Overall, our main contributions are primarily twofold:

(1) The super-efficiency SBM model based on unintended output is used to quantify eco-efficiency and new urbanization efficiency, and the input-output index system is more comprehensive, scientific, and reasonable. In the indicator system for unexpected output, carbon emissions are included with conventional industrial wastes. This inclusion is consistent with the "double carbon" objective and enables more comprehensive and accurate calculation results that reflect the meaning and reality of the development of modern urbanization and ecological civilization, and the super-efficiency can solve the problem that the traditional DEA model cannot accurately distinguish effective decision-making units.

(2) This research examines the spatial-temporal distribution pattern and regional disparities of the coupling and coordinated growth of new urbanization efficiency and eco-efficiency, using Fujian Province as an example. In addition, a spatial econometric model is developed to quantify the interaction effect and mechanism of new urbanization efficiency and eco-efficiency. This analysis aims to give countermeasures, recommendations, and points of reference for the development of modern urbanism and ecological civilization in Fujian Province and other provinces.

## 2. Study area and methods

### 2.1. Study area

**Fig 1:**
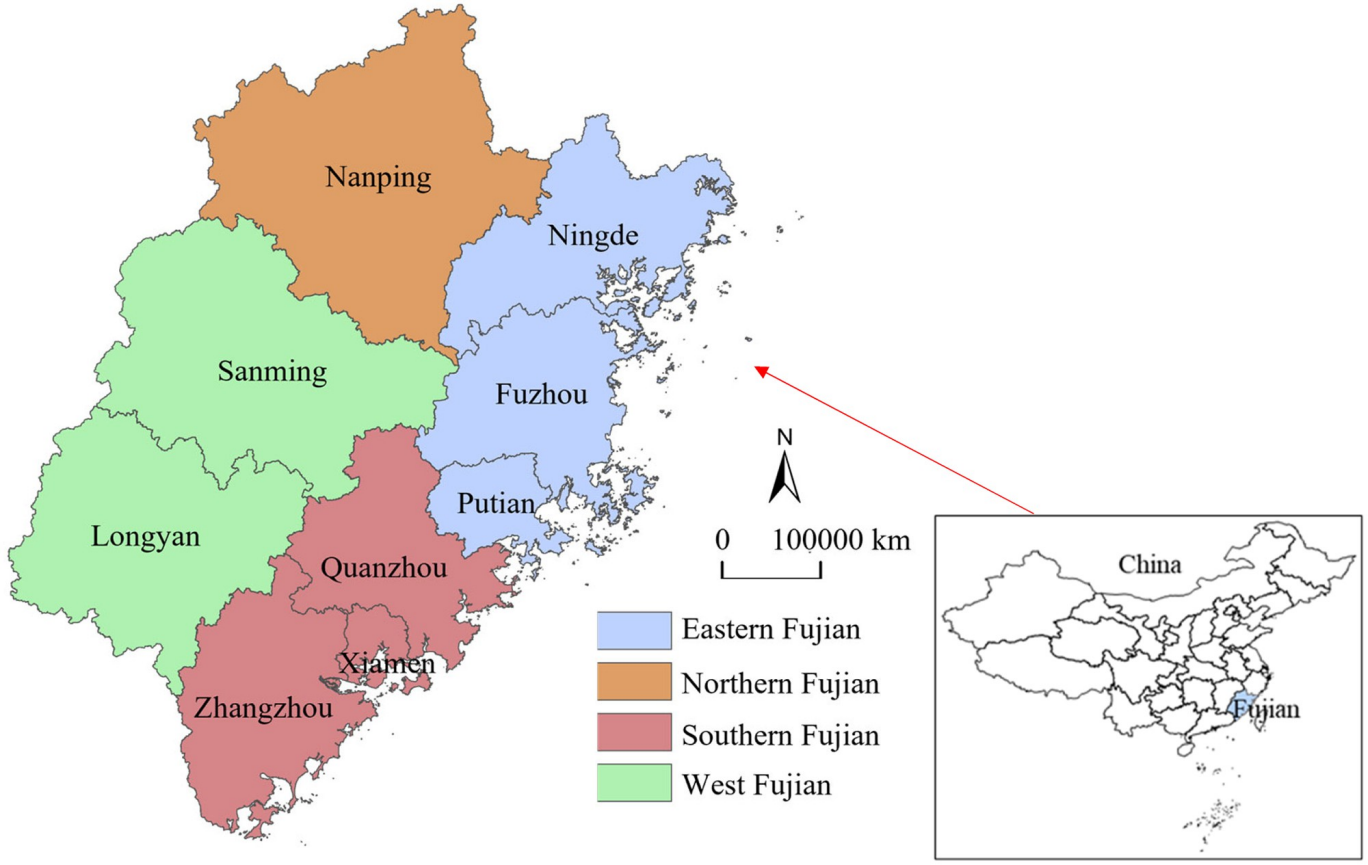
Location of study area. (Reprinted from ArcGIS under a CC BY license, original copyright 2023).

Fujian is situated on the coast of the East China Sea in the southeast of China. Between 23°33′ N and 28°20′ N, and 115°50′ E and 120°40′ E, its landmass is located. It links the Yangtze Delta with the Pearl Delta. It is the starting point of the Maritime Silk Road and Zheng He’s expeditions to the West, as well as a key communication window and hub for China. As shown in Fig 1, Fujian province has authority over Fuzhou (including Pingtan Comprehensive Experimental Zone), Xiamen, Zhangzhou, Quanzhou, Sanming, Putian, Nanping, Longyan, and Ningde, a total of 9 districts and cities with a land size of 124000 km^2^ and a sea area of 136000 km^2^. As one of the first national ecological civilization demonstration zones in China, Fujian Province has long been committed to creating green economy and low-carbon circular economy, and ecological environment indicators are of a reasonably high quality. Yet, the economic foundations of different cities in Fujian Province are unequal, owing to substantial variations in natural resources and land shape. By the end of 2021, the urbanization rate of Fujian Province’s permanent population has reached 69.7%, ranking it first in the nation. “The New Urbanization Plan of Fujian Province (2021–2035)” indicates that the new urbanization will be substantially completed by 2035, with the two metropolitan areas of Fujian Province serving as the main engines driving the development of the Guangdong, Fujian, and Zhejiang coastal urban agglomeration.

### 2.2. Research methods

#### 2.2.1. Index system construction and data source

Referencing relevant research [3, 22], this paper defines the new urbanization efficiency as the proportional relationship between the output benefits (population benefits, economic benefits, social benefits, residential space benefits, and ecological benefits) of a city (or region) and the various factors (land, labor, capital, technology, resources, energy, etc.) that need to be invested in the process of urbanization over a certain time period. Eco-efficiency should be defined as: in the resource-environment-economic system, the ratio of the economic benefits achieved and the negative benefits generated on resources and environment to the actual input of various factors in economic production activities, which reflects the utilization efficiency and impact of economic development on resources and environment.

The input-output index system is created according to the criteria of representativeness, conciseness, scientific rigor, and systematization, using the input-output theory and production function model as a foundation. According to current research, the input indicators focus primarily on land, capital, and labor, while also considering the input of resources and energy. Indicators of output primarily include economic and ecological advantages. Under the new urbanization efficiency evaluation index system, the coordinated development of population, economics, science and technology, resources, and the natural environment is emphasized. The input index emphasizes the incorporation of “real utilization of foreign investment” and “percentage of R&D internal expenditure to public budget expenditure.” To reflect the process and outcomes of new urbanization, the expected output is selected from the five aspects of population, economy, society, space, and ecology. Regarding social urbanization, the per capita disposable income of urban and rural residents is chosen as the representative, and the per capita disposable income of urban and rural residents is computed using a weighted average with the urbanization rate serving as the weight. In the eco-efficiency indicator system, the input of natural factors such as water, energy, and land is given greater importance. In addition to economic development and ecological benefits, the expected output also contains circular economic advantages. In consideration of the potential availability of indicators, the centralized treatment rate of sewage treatment plants is chosen for classification. To avoid the impact of too many indicators on the efficiency measurement results, the traditional industrial “three wastes” are combined into one indicator—“industrial pollutant emissions” in the undesired output of the two indicator systems, and based on the “Carbon peaking and carbon neutrality” goals, “carbon emissions” is taken as a component of the undesired output, which compensates the flaw of ignoring carbon emissions in the existing research. Carbon emissions are the total amount of carbon dioxide emissions generated by various primary energy consumption. The calculation method mainly refers to the relevant research of Zhou *et al*. [23]. Specific indicators are shown in Table 1.

**Table 1:** Input-output index system of new urbanization efficiency and eco-efficiency.

	Indicator properties	Indicator category	Specific indicators	Unit
New urbanization efficiency	Input of factors	Land input	Land area for construction	km^2^
		Labor input	Number of non-agricultural employed persons	10^4^ person
		Capital input	Fixed asset investment in urban areas	10^4^ Yuan
			Actual utilization of foreign direct investment	10^4^ USD
		Technology input	Proportion of R&D internal expenditure to public budgetary expenditure	%
		Resource input	Total annual water supply	10^4^ m^3^
		Energy input	Total electricity consumption in the society	10^4^ W·h
	Expected output	Population urbanization	Urban population	10^4^ person
		Economic urbanization	Output value of the secondary and tertiary industries	10^8^ Yuan
		Social urbanization	Per capita disposable income of urban and rural residents	Yuan
		Spatial urbanization	Proportion of built-up area to total land area	%
		Ecological urbanization	Green coverage rate in built-up areas	%
	Unexpected output	Ecological negative externality	Industrial pollutant emissions	10^4^ metric tons
			Carbon emissions	10^4^ metric tons
Eco-efficiency	Input of factors	Capital investment	Total fixed asset investment	10^8^ Yuan
		Labor input	Employment numbers	10^4^ person
		Land input	Built-up area	km^2^
		Energy input	Total energy consumption	10^4^ tons of standard coal
		Water resource input	Annual total water supply	10^4^ m^3^
	Expected output	Economic development benefits	GDP	10^8^ Yuan
		Circular economy benefits	Centralized treatment rate of sewage treatment plants	%
		Ecological benefits	Per capita green area	hm^2^
	Unexpected output	Ecological negative effects	Industrial pollutant emissions	10^4^ metric tons
			Carbon emissions	10^4^ metric tons

In consideration of the consistency and the quality of the data, except that the carbon emissions are calculated, other indicator data mainly come from the “CHINA CITY STATISTICAL YEARBOOK” from 2011 to 2021, and the missing data are filled using linear interpolation method.

#### 2.2.2. Undesired output super-efficiency SBM model

Modern production mode produces a vast number of unanticipated outputs, including waste water, waste gas, waste residue, and additional carbon emissions, while simultaneously improving labor productivity and socio-economic level. Based on Tone’s research [24], the super-efficiency SBM model of unexpected output is created to quantify the new urbanization’s efficiency and eco-efficiency. The model is non-radial and non-oriented, which can effectively resolve the difference of results caused by the selection of radial and angle in the traditional DEA model and the problems caused by the slack variables [25], as well as effectively distinguish the evaluation units located at the frontier, so as to further clarify the difference of efficiency levels between cities. The model expression is as follows:

$$\begin{array}{l} {\text{min}}_{\rho }=\frac{1+\frac{1}{m}\sum_{i=1}^{m}s_{i}^{-}/x_{ik}}{1-\frac{1}{q_{1}+q_{2}}(\sum_{r=1}^{q_{1}}\frac{s_{r}^{+}}{y_{r0}}+\sum_{l=1}^{q_{2}}\frac{s_{l}^{b-}}{z_{l0}})}\\ s.t.\left\{\begin{array}{l} \sum_{j=1,j\neq j_{0}}^{n}x_{j}\lambda_{j}-s_{i}^{-}\leq x_{i0}(i=1,2,\ldots ,m)\\ \sum_{j=1,j\neq j_{0}}^{n}y_{j}\lambda_{j}+s_{r}^{+}\geq y_{r0}(r=1,2,\ldots ,q_{1})\\ \sum_{j=1,j\neq j_{0}}^{n}z_{j}\lambda_{j}-s_{l}^{b-}\leq z_{l0}(l=1,2,\ldots ,q_{2})\\ 1-\frac{1}{q_{1}+q_{2}}(\sum_{r=1}^{q_{1}}\frac{s_{r}^{+}}{y_{r0}}+\sum_{l=1}^{q_{2}}\frac{s_{l}^{b-}}{z_{l0}})>0\\ \lambda_{j}\geq 0,s_{i}^{-}\geq 0,s_{r}^{+}\geq 0,s_{l}^{b-}\geq 0,(j=1,2,\ldots ,n,j\neq j_{0}) \end{array}\right. \end{array}$$ (1)

where, *ρ* is the efficiency value; if it is less than 1, the decision-making unit (DMU) is invalid, and if it is more than or equal to 1, the DMU is effective. The larger the value, the higher the efficiency. $s_{i}^{-}$, $s_{r}^{+}$, $s_{l}^{-}$ represent the input, expected output, and unexpected output slack variables, respectively. ***m***, ***q***_1_, ***q***_2_ represent the number of input variables, expected output, and unexpected output, respectively. *x*_*j*_, *y*_*j*_, *z*_*j*_ are the values of input, expected output, and unexpected output variables of the ***j***_***th***_ DMU, respectively. *x*_***i*0**_, *y*_*r*0_, *z*_**l0**_ are the values of the input, expected output and unexpected output variables of the evaluated unit. *λ*_*j*_ is the weight vector. *n* is the number of DMUs.

#### 2.2.3. Coupling coordination model

The coupling coordination model is extensively utilized in the study of the coordination relationships between various entities. Coupling refers to the degree of system interaction, while coordination refers to the degree of benign coupling in the interaction; together, they influence the system’s evolution. New urbanization efficiency and eco-efficiency are two independent systems that interact with one another. In this study, a coupling coordination model is constructed to investigate the coupling and coordination development of the two entities. The model is expressed as follows:

$$C=2\times {\left[\frac{E_{1}\cdot E_{2}}{{\left(E_{1}+E_{2}\right)}^{2}}\right]}^{\frac{1}{2}}$$ (2)

$$D=\sqrt{C\cdot (\alpha E_{1}+\beta E_{2})}$$ (3)

where, *C* is the coupling, which is divided according to the standard of Yin *et al*. [12]; *D* is the coupling coordination scheduling, with a value range of (0,1), which is divided into 10 levels at 0.1 intervals. The degrees of coupling coordination are classified as follows: extreme disorder, severe disorder, moderate disorder, mild disorder, near disorder, reluctant coordination, primary coordination, intermediate coordination, good coordination, and high-quality coordination. A higher value of *D* indicates a better degree of coupling coordination. *E*_**1**_ and *E*_2_ represent new urbanization efficiency and eco-efficiency, respectively. The weights of new urbanization efficiency and eco-efficiency are denoted by *α* and *β*, and *α* + *β* = 1. This paper considers both measures equally important and thus sets *α* = *β* = 0.5.

#### 2.2.4. The convergence coefficient *σ*

The convergence coefficient *σ* can be used to access the evolution trend of the development gap between different regions [26]. It can also be used to analyze the spatial equilibrium effect of new urbanization efficiency, eco-efficiency, and dual efficiency coupling and co-scheduling. If the coefficient decreases over time, indicating *σ* convergence, this suggests that there is a spatial equalization effect. Conversely, if the coefficient remains stable or increases, then spatial differentiation will become more evident. The specific formula for the convergence coefficient is as follows:

$$\sigma_{t}=\frac{1}{{\bar{E}}_{t}}\sqrt{\frac{1}{n}\sum_{i=1}^{n}{\left(E_{it}-\bar{E_{t}}\right)}^{2}}$$ (4)

Where *σ*_*t*_ represents the convergence coefficient of an index in year *t*, $\bar{E_{t}}$ is the mean value of an index in each region in year *t*, and *n* denotes the number of regions.

#### 2.2.5. Spatial autocorrelation test

*Morans’s I* is a widely used method for testing spatial autocorrelation. In this study, we employ the univariate global spatial autocorrelation test [27, 28] to analyze the spatial correlation of new urbanization efficiency, eco-efficiency, and dual efficiency coupling co-scheduling in Fujian Province. The specific formula for *Morans’s I* is as follows:

$$Morans’s\;I=\frac{\sum_{i=1}^{n}\sum_{j=1}^{n}w_{ij}\left(E_{i}-\bar{E}\right)\left(E_{j}-\bar{E}\right)}{\frac{1}{n}\sum_{i=1}^{n}{\left(E_{i}-\bar{E}\right)}^{2}\sum_{i=1}^{n}\sum_{j=1}^{n}w_{ij}}$$ (5)

where *Morans’s I* is the univariate global spatial autocorrelation coefficient, *E*_*i*_ and *E*_*j*_ are the new urbanization efficiency or eco-efficiency of the *i*_*th*_ and *j*_*th*_ space units, respectively. $\bar{E}$ represents the average value of new urbanization efficiency or eco-efficiency in all space units. *n* is the number of space units, and *w*_*ij*_ is the spatial weight matrix constructed based on the reciprocal of Euclidean distance between different space units.

## 3. Results

### 3.1. Temporal variation characteristics of new urbanization efficiency and eco-efficiency

**Fig 2:**
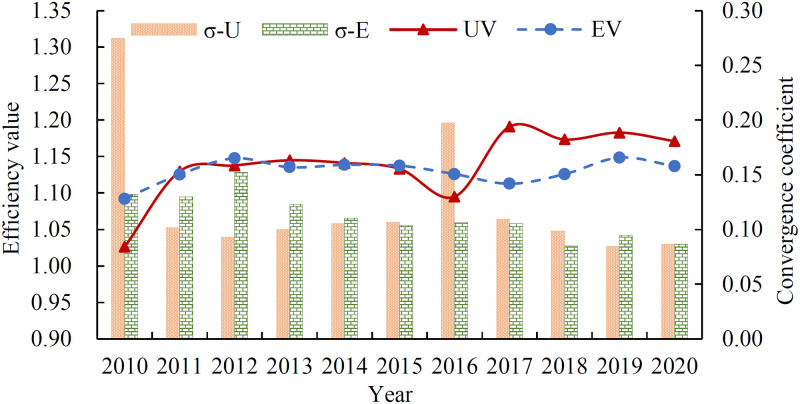
Overall trend of new urbanization efficiency and eco-efficiency in Fujian Province. Note: σ-U: Convergence coefficient of new urbanization efficiency; σ-E: Convergence coefficient of eco-efficiency; UE: New urbanization efficiency; EE: Eco-efficiency. Same as below.

Based on the undesirable super-efficiency SBM model, this work calculates the new urbanization efficiency and eco-efficiency of nine cities in Fujian Province from 2010 to 2020 using the MATLAB R2019a software. The results are presented in Tables 2 and 3. Additionally, the change trend of the efficiency data for the nine cities each year is illustrated in Fig 2, which displays the yearly averages of efficiency data for the cities. As shown in Fig 2, the overall new urbanization efficiency and eco-efficiency of Fujian Province have reached an effective level, with changes over time that can be divided into three distinct stages. In the first stage (2010–2011), the new urbanization efficiency was lower than eco-efficiency, and then both measures increased rapidly, achieving equivalent efficiency by 2011. During the second stage (2012–2016), both new urbanization efficiency and eco-efficiency exhibited a pattern of “mutual restraint and decline,” with both measures exhibiting a fluctuating downward trend. By 2016, new urbanization efficiency had reached its lowest point, declining by 3.01% compared to 2011, while the rate of decline in eco-efficiency was relatively slow. In the third stage (2017–2020), new urbanization efficiency rapidly increased to its highest value of 1.19 in 2017, then fluctuated and decreased. Meanwhile, eco-efficiency reached its lowest point of 1.11 in 2017 and then increased and decreased. Throughout this stage, new urbanization efficiency consistently surpassed eco-efficiency, with the difference between the two remaining stables at about 0.35 since 2018, showing similar trends of change. The convergence coefficient *σ* of new urbanization efficiency in Fujian Province was relatively large in 2011 and 2016, indicating a significant gap in the level of new urbanization between cities in those years. However, the coefficient showed a decreasing trend after 2016, indicating that the difference in the level of new urbanization between regions was well controlled. During the entire study period, the convergence coefficient *σ* of eco-efficiency showed an initially increasing trend and then a decreasing trend, indicating that the level of ecological environment in various cities in Fujian Province exhibited a spatial equilibrium effect.

**Table 2:** New urbanization efficiency value of each city in Fujian Province from 2010 to 2020.

Year	FZ	XM	PT	SM	QZ	ZZ	NP	LY	ND
2010	1.22	1.21	1.02	1.03	1.25	1.13	0.53	0.52	1.32
2011	1.30	1.26	1.02	1.04	1.29	1.13	1.01	1.00	1.12
2012	1.31	1.26	1.01	1.06	1.22	1.18	1.03	1.02	1.14
2013	1.32	1.31	1.02	1.06	1.22	1.19	1.07	1.00	1.11
2014	1.33	1.32	1.01	1.07	1.24	1.14	1.04	1.02	1.09
2015	1.34	1.32	1.02	1.08	1.23	1.11	1.03	1.02	1.07
2016	1.30	1.30	1.02	1.10	1.25	1.11	1.06	0.55	1.17
2017	1.40	1.35	1.19	1.13	1.32	1.20	1.09	1.02	1.02
2018	1.14	1.37	1.08	1.22	1.37	1.15	1.12	1.02	1.09
2019	1.11	1.36	1.09	1.15	1.34	1.16	1.14	1.06	1.23
2020	1.12	1.33	1.13	1.24	1.33	1.14	1.16	1.03	1.07
Mean	1.26	1.31	1.05	1.11	1.28	1.15	1.03	0.93	1.13
Ranking	3	1	7	6	2	4	8	9	5

Note: FZ: Fuzhou; XM: Xiamen; PT: Putian; SM: Sanming; QZ: Quanzhou; ZZ: Zhangzhou; NP: Nanping; LY: Longyan; ND: Ningde. The same as below.

**Table 3:** Eco-efficiency value of each city in Fujian Province from 2010 to 2020.

Year	FZ	XM	PT	SM	QZ	ZZ	NP	LY	ND
2010	1.05	1.50	1.04	1.03	1.06	1.03	1.02	1.05	1.05
2011	1.16	1.48	1.07	1.04	1.05	1.01	1.02	1.05	1.26
2012	1.18	1.47	1.06	1.03	1.05	1.00	1.04	1.04	1.45
2013	1.17	1.46	1.07	1.03	1.08	1.00	1.06	1.05	1.29
2014	1.25	1.42	1.06	1.03	1.16	1.01	1.09	1.05	1.17
2015	1.24	1.42	1.08	1.05	1.13	1.03	1.06	1.06	1.16
2016	1.27	1.40	1.09	1.04	1.11	1.04	1.06	1.02	1.10
2017	1.19	1.38	1.17	1.06	1.13	1.04	1.06	1.04	0.95
2018	1.22	1.35	1.16	1.06	1.12	1.06	1.05	1.04	1.08
2019	1.17	1.41	1.15	1.04	1.09	1.05	1.05	1.21	1.17
2020	1.17	1.36	1.11	1.04	1.09	1.04	1.05	1.23	1.14
Mean	1.19	1.42	1.10	1.04	1.10	1.03	1.05	1.08	1.17
Ranking	2	1	5	8	4	9	7	6	3

**Fig 3:**
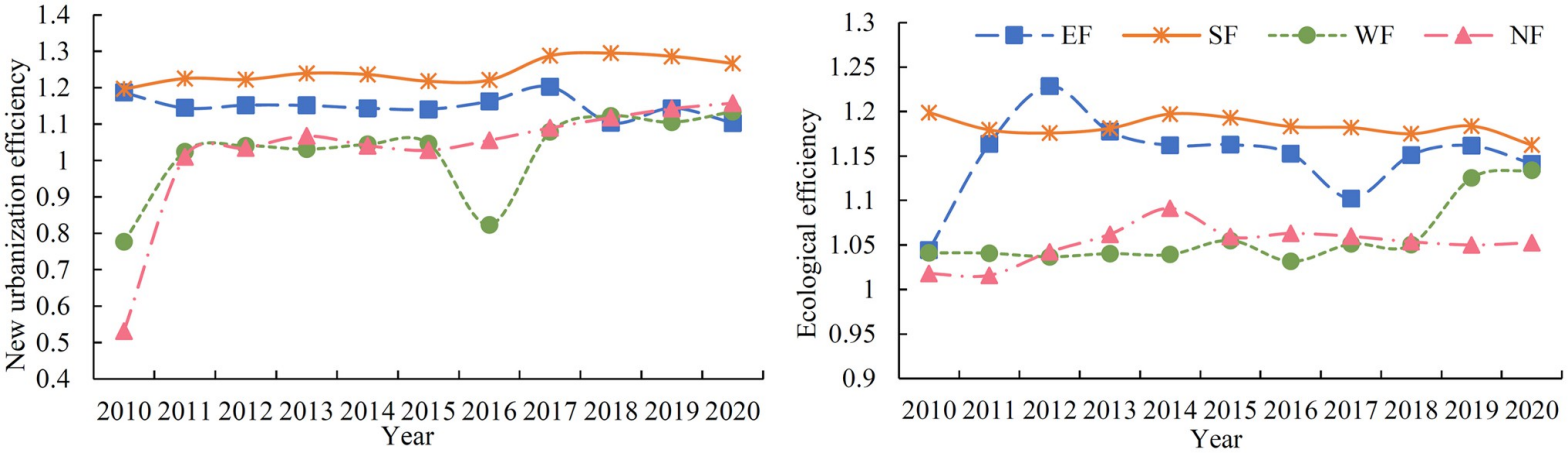
Trends of new urbanization efficiency and eco-efficiency in each region of Fujian Province. Note: EF: Eastern Fujian; WF: Western Fujian; SF: Southern Fujian; NF: Northern Fujian.

Based on Fig 3, it’s clear that both new urbanization efficiency and eco-efficiency were much higher in southeast Fujian than they were in northwest Fujian. Moreover, the new urbanization efficiency in southern Fujian (Xiamen, Quanzhou, and Zhangzhou) exceeded eco-efficiency, while in other regions the opposite was true. Regarding new urbanization efficiency, it was ineffective in northern Fujian (Nanping) and western Fujian (Longyan and Sanming) in 2010, but it has increased each year since 2011. Except for the steep decline in western Fujian (mainly Longyan) in 2016, new urbanization efficiency has been effective in other periods. Meanwhile, the new urbanization efficiency of eastern Fujian (Fuzhou, Ningde, and Putian) and southern Fujian remained relatively stable, with a slight decline from 2017. With respect to eco-efficiency, all regions showed effective efficiency from 2010 to 2020, but southeast Fujian’s efficiency value was 0.1 higher on average than that of northwest Fujian. The eco-efficiency of eastern Fujian exhibited significant fluctuations, while that of western Fujian has improved considerably in recent years, increasing by 8% from 2018 to 2020, while other regions showed a slight downward trend.

### 3.2. Spatial distribution characteristics of new urbanization efficiency and eco-efficiency

**Fig 4:**
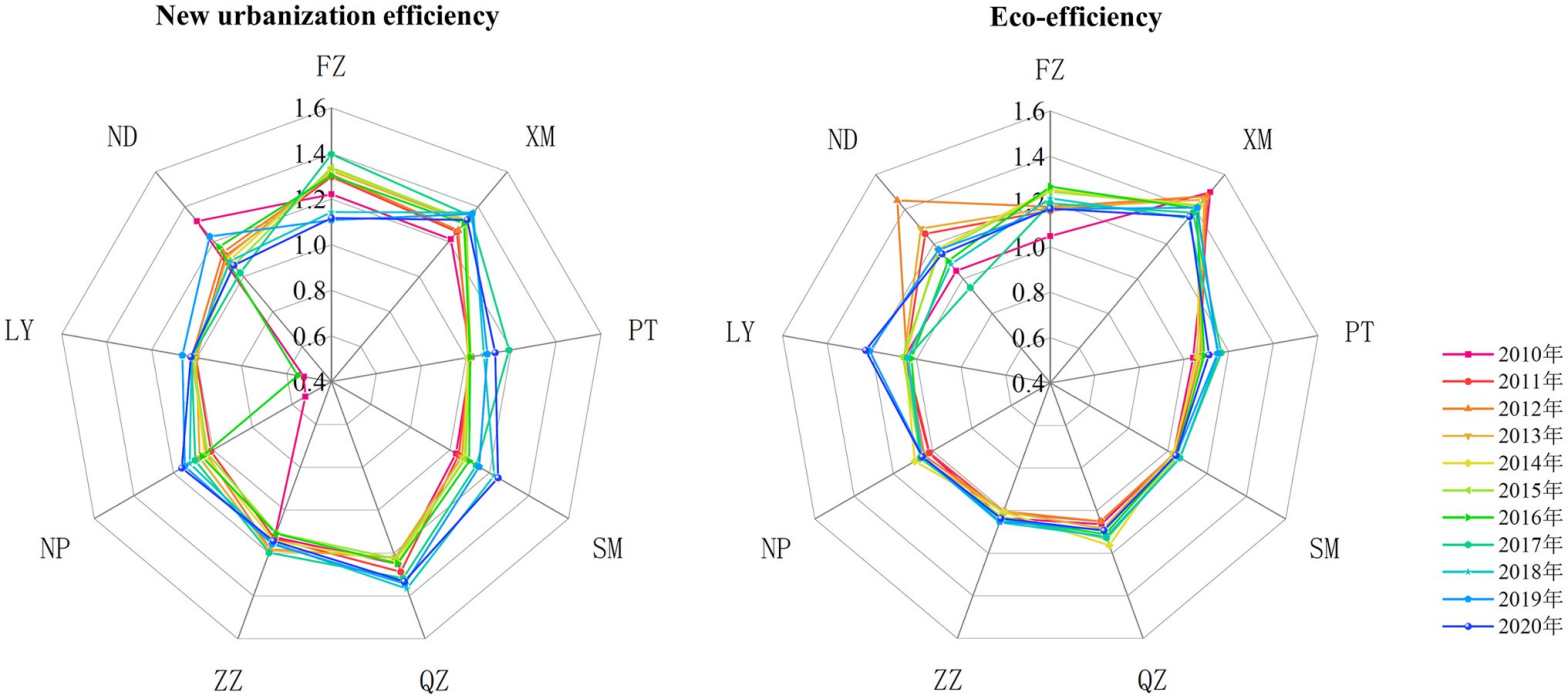
The trend of new urbanization efficiency and eco-efficiency in each city of Fujian Province.

Fig 4 shows that “dynamic differentiation of dual efficiency” exists in different parts of Fujian Province. Generally, the eco-efficiency of each region showed slight fluctuations during the study period, while new urbanization efficiency underwent significant changes. Xiamen consistently ranked highest in dual efficiency levels, with its eco-efficiency slightly higher than new urbanization efficiency, indicating a stable DMU with “high-high” dual efficiency. However, in recent years, the dual efficiency trend has declined steadily. According to Tables 2 and 3, Quanzhou ranked second in new urbanization efficiency and fourth in eco-efficiency, with both exhibiting reverse changes over time. That is, new urbanization efficiency increases while eco-efficiency first increases and then decreases, representing a changing DMU with “high-medium” dual efficiency. Fuzhou’s dual efficiency level was second only to Xiamen, exhibiting significant changes in range, with both showing a trend of rising first and then falling, representing a changing DMU with “high-high” dual efficiency. Ningde’s dual efficiency showed significant fluctuations, particularly in eco-efficiency, from 2011 to 2014, resulting in a higher average eco-efficiency. In recent years, both have tended to stabilize, representing a changing DMU with “medium-medium” dual efficiency. The new urbanization efficiency of Sanming, Nanping, Putian, and Zhangzhou has increased year by year, while eco-efficiency has only slightly increased. Overall, new urbanization efficiency is higher than eco-efficiency, indicating a stable DMU with “low-low” dual efficiency. Conversely, Longyan exhibited relatively stable new urbanization efficiency, while eco-efficiency showed rapid improvement, representing a changing DMU with “low-high” dual efficiency.

**Fig 5:**
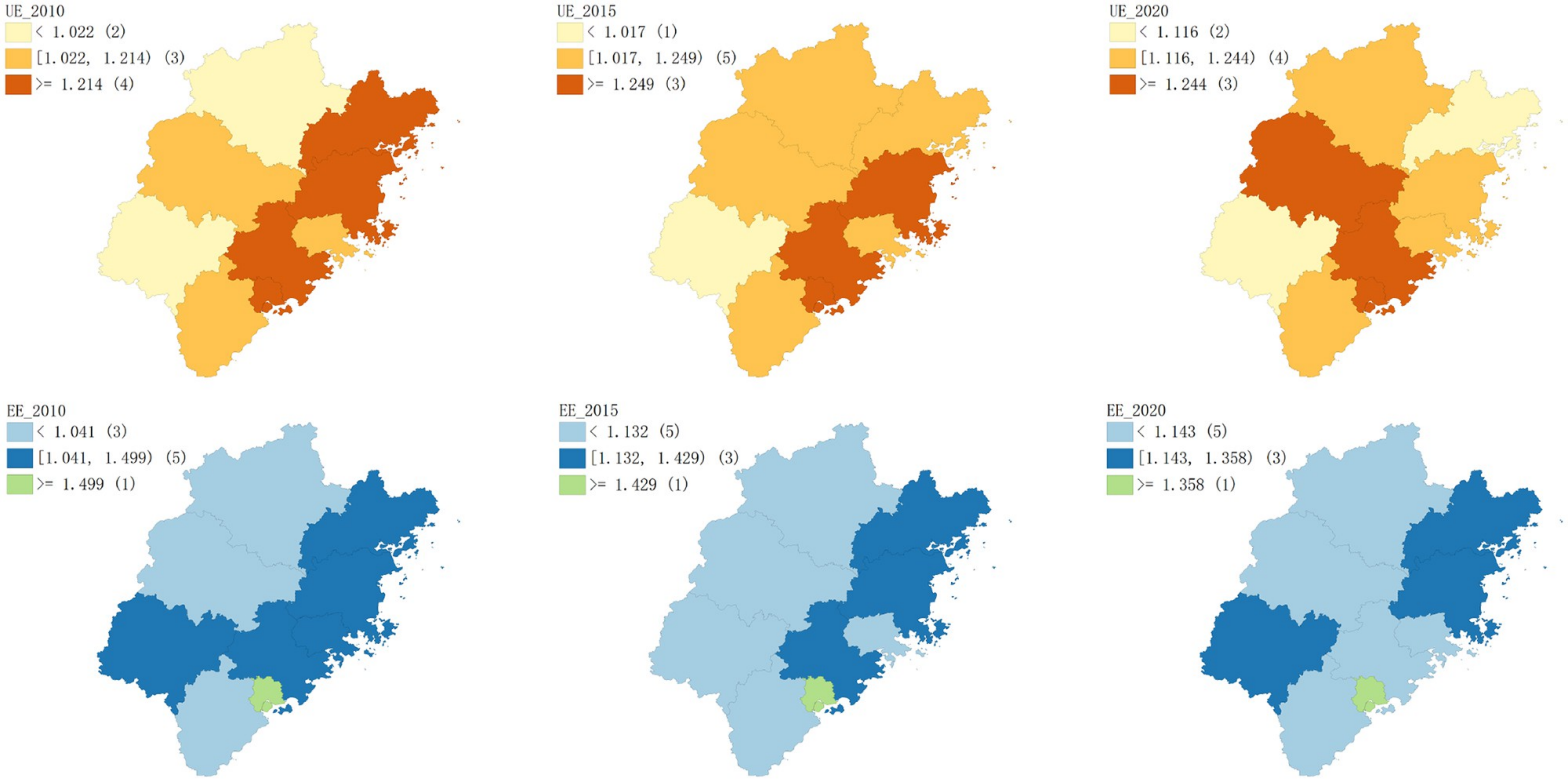
Spatial distribution characteristics of new urbanization efficiency and eco-efficiency in each city of Fujian Province. (Reprinted from ArcGIS under a CC BY license, original copyright 2023).

**Fig 6:**
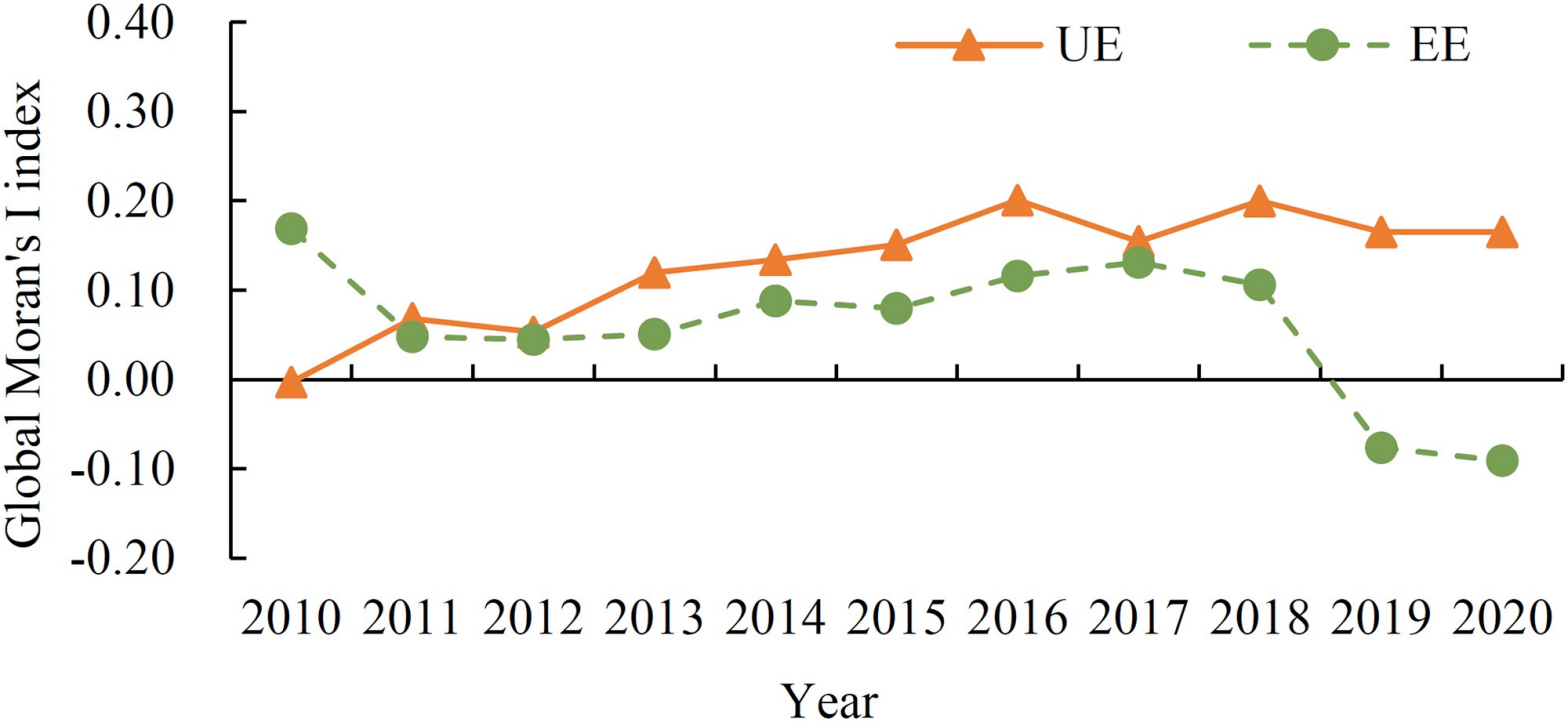
The global Moran’s I index of new urbanization efficiency and eco-efficiency in Fujian Province.

The spatio-temporal distribution pattern of new urbanization efficiency and eco-efficiency in Fujian Province was further analyzed using GeoDa software to draw the spatial distribution map of natural breakpoints for dual efficiency in 2010, 2015, and 2020 (Fig 5). The single variable global Moran’s I was then utilized to measure the spatial correlation characteristics of new urbanization efficiency and eco-efficiency, with the results depicted in Fig 6. The spatial distribution map in Fig 5 reveals that the new urbanization efficiency in Xiamen and Quanzhou has consistently been high, while that in Zhangzhou and Putian has remained at a medium level. Longyan, on the other hand, has had consistently low new urbanization efficiency, whereas Sanming and Nanping have shown a gradual increase, while Ningde has exhibited a decline from the high efficiency zone in 2010 to the low efficiency zone in 2020. Fuzhou has also experienced a slight decrease in new urbanization efficiency. Overall, the spatial pattern of new urbanization efficiency in Fujian Province has undergone significant changes. By contrast, the spatial pattern of eco-efficiency has changed only slightly. Xiamen’s eco-efficiency remains far ahead, with Fuzhou in second place. Ningde’s eco-efficiency has remained at a medium level, while Sanming, Nanping, and Zhangzhou’s have been relatively low. Longyan’s eco-efficiency has fluctuated greatly, reaching a medium efficiency level by 2020, while Quanzhou and Putian’s eco-efficiency has shown a downward trend, declining from the medium efficiency level in 2010 to the low efficiency level in 2020. Fig 6 indicates that during the study period, the dual efficiency of Fujian Province displayed a positive spatial autocorrelation. High new urbanization efficiency (or eco-efficiency) areas have played a positive role in promoting the development of new urbanization (ecological environment) in surrounding regions, and the spatial agglomeration effect of new urbanization efficiency has been higher than that of eco-efficiency. From 2019, however, the Moran’s I of eco-efficiency has shifted from positive to negative, indicating a weakening of the spatial agglomeration effect.

### 3.3. Spatio-temporal distribution characteristics of the coupling and coordinated development of new urbanization efficiency and eco-efficiency

#### 3.3.1. Analysis of overall dual efficiency coupling and coordinated development trend

Table 4 reveals that the overall dual efficiency coupling degree and coordination degree in Fujian Province exhibit a fluctuating trend. Specifically, the coupling degree went through a cyclical pattern of “run-in→high-level coupling→run-in→high-level coupling→run-in”. The highest coupling degree was observed in 2017, reaching 0.92, after which it decreased year by year. On the other hand, the coupling coordination degree was not high, averaging 0.5 throughout the years, and underwent a staggered cycle of “near disorder and reluctant coordination”. Until 2020, the dual efficiency barely reached coordination, indicating that there is still a certain gap with primary coordination. Furthermore, the convergence coefficient *σ* of the coupling coordination degree of the dual efficiency in each city decreased year after year, implying that the gap between the coupling coordination development of the dual efficiency among regions is gradually closing, demonstrating the spatial equalization effect.

**Table 4:** Coupling coordination condition of new urbanization efficiency and eco-efficiency in Fujian Province from 2010 to 2020.

Year	C	Degree of coupling	D	Degree of coupling coordination	Convergence coefficient of coupling coordination	Global Moran’I of coupling coordination
2010	0.62	run-in phase	0.45	near disorder	0.510	0.178**
2011	0.79	run-in phase	0.46	near disorder	0.586	0.162*
2012	0.79	run-in phase	0.49	near disorder	0.547	0.073
2013	0.80	run-in phase	0.51	reluctant coordination	0.502	0.16**
2014	0.82	high-level coupling phase	0.51	reluctant coordination	0.543	0.144**
2015	0.80	run-in phase	0.47	near disorder	0.603	0.148**
2016	0.79	run-in phase	0.59	reluctant coordination	0.425	0.276**
2017	0.92	high-level coupling phase	0.57	reluctant coordination	0.465	0.176**
2018	0.87	high-level coupling phase	0.51	reluctant coordination	0.467	0.201**
2019	0.70	run-in phase	0.48	near disorder	0.482	0.208**
2020	0.69	run-in phase	0.50	reluctant coordination	0.429	0.146**

Note:C represents the degree of coupling, and D represents the degree of coupling coordination.

*and ** denote significance at the level of 5%, and 1% respectively.

#### 3.3.2. The spatio-temporal distribution characteristics of the coupling and coordinated development of dual efficiency in various regions

The analysis shown in Table 5 shows that coupling and coordinated development of dual efficiency has changed a lot over time in different cities in Fujian Province. Xiamen has consistently maintained high-quality coordination in its dual efficiency, while Quanzhou has shown a development trend from reluctant coordination to primary coordination. Fuzhou’s dual-efficiency coordination level has been highly fluctuating, with a gradual increase from 2010 to 2017, followed by a decline until barely coordinated in 2020. Putian’s dual efficiency was in an imbalanced state before 2015 and reached a barely coordinated level in 2016. The coupling coordination degree of Ningde dual efficiency exhibits a downward trend, transitioning from coordination to disorder. The dual efficiency of Sanming, Zhangzhou, Nanping, and Longyan has predominantly been in a state of imbalance throughout the study period, with slight improvements in the coupling coordination degree of Nanping and Sanming in recent years, while the disorder degree of Longyan and Zhangzhou has remained high. To further examine the relationship and difference between the coupling coordination degree of new urbanization efficiency and eco-efficiency in different regions, the K-Means clustering method was applied to divide the nine cities in Fujian Province into three gradients based on their coupling coordination degree of dual efficiency between 2010 and 2020. According to the findings, Xiamen represents the highly coupled and coordinated dual efficiency development zone, with an average class center of 0.98. Fuzhou, Quanzhou, and Ningde belong to the moderate coupling and coordinated development zone of dual efficiency with an average class center of 0.64, while Putian, Zhangzhou, Longyan, Sanming, and Nanping are part of the low coupling and coordinated development zone of dual efficiency with an average class center of only 0.33. The analysis reveals that the coupling and coordinated development level of new urbanization efficiency and eco-efficiency in various cities in Fujian Province demonstrate significant regional differences, with high coupling and coordination areas mostly concentrated in the southeast of Fujian, while the coupling and coordination level in the northwest of Fujian is generally low.

**Table 5:** Coupling coordinated development of new urbanization efficiency and eco-efficiency in Fujian Province from 2010 to 2020.

Year	FZ	XM	PT	SM	QZ	ZZ	NP	LY	ND
2010	ND	HC	ND	ID	RC	ND	SD	SD	RC
2011	MC	HC	MD	ID	RC	MD	SD	SD	PC
2012	MC	HC	SD	ID	RC	MD	ID	MD	GC
2013	MC	HC	ID	ID	RC	MD	ND	SD	PC
2014	GC	HC	SD	ID	MC	MD	ID	MD	RC
2015	GC	HC	MD	ID	PC	MD	MD	SD	ND
2016	GC	HC	RC	ND	PC	ND	RC	SD	PC
2017	GC	HC	PC	RC	MC	RC	ND	MD	SD
2018	PC	HC	RC	ND	MC	ID	ID	SD	ND
2019	ND	HC	ND	MD	PC	ID	ID	MD	PC
2020	RC	HC	RC	ID	PC	MD	ND	MD	ND

Note: ED: Extreme Disorder; SD: Severe Disorder; MD: Moderate Disorder; ID: Mild Disorder; ND: Near Disorder; RC: Reluctant Coordination; PC: Primary Coordination; MC: Intermediate Coordination; GC: Good Coordination; HC: High-quality Coordination.

#### 3.3.3. Analysis of spatial agglomeration characteristics of the coupling coordination degree of dual efficiency

**Fig 7:**
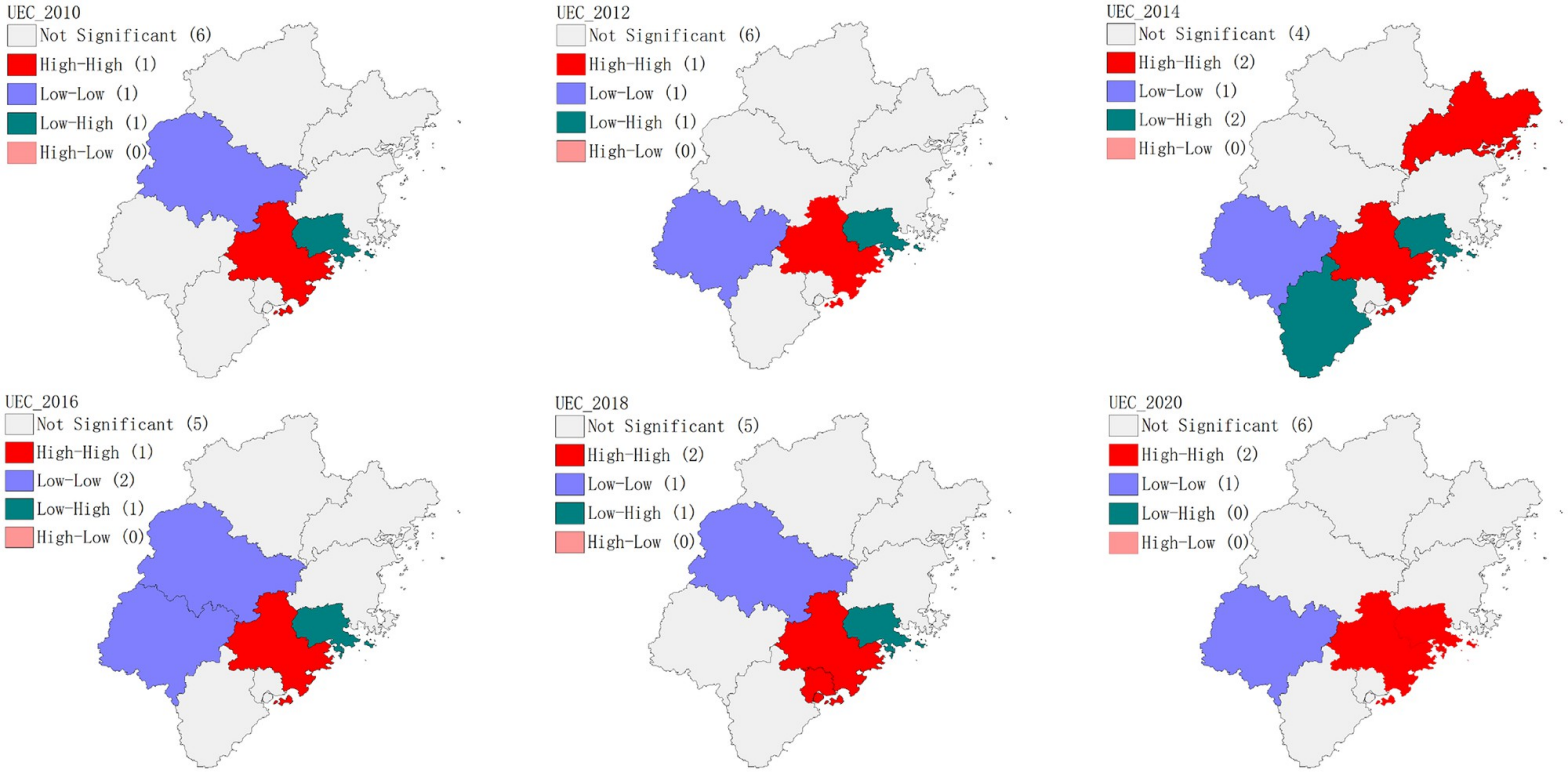
Spatial LISA agglomeration of coupling coordination of new urbanization efficiency and eco-efficiency in Fujian Province. Note: UEC: Coupling coordination of new urbanization efficiency and eco-efficiency. (Reprinted from ArcGIS under a CC BY license, original copyright 2023).

Table 4 shows that the global Moran’s I of the coupling coordination degree of dual efficiency between 2010 and 2020 is positive, with all other years except 2012 being statistically significant. This suggests that there is a positive spatial correlation between the coupling coordination degree of dual efficiency in all regions of Fujian Province, and the spatial agglomeration effect is significant. Using the local Moran index, GeoDa software was used to further draw the spatial LISA aggregation diagram of the coupling coordination degree of dual efficiency, as depicted in Fig 7. The diagram identifies three types of spatial distribution during the research period: “low-low” agglomeration, “low-high” agglomeration, and “high-high” agglomeration. The “low-low” concentration is mainly distributed in the northwest of Fujian, with Sanming, Longyan, and Nanping having a low coupling coordination degree of dual efficiency, and the spatial difference being relatively small. The “low-high” concentration is mainly concentrated in Zhangzhou and Putian, with Putian’s coupling coordination degree of new urbanization efficiency and eco-efficiency being much lower than the surrounding cities throughout the research period, and thus remaining in the depression of regional development. The spatial difference of the coupling coordination degree of dual efficiency between Zhangzhou and its surrounding areas is large, and much lower than that of Quanzhou and Xiamen. Quanzhou has consistently shown the characteristics of “high-high” concentration, with a high degree of coupling coordination of dual efficiency and a small spatial difference with Xiamen. In 2018, Xiamen also demonstrated a significant “high-high” concentration feature, indicating that Quanzhou and Xiamen have achieved mutual benefit in the development of new urbanization and the construction of ecological civilization. Moreover, Putian benefited from the spatial spillover effect of Quanzhou, and showed the “high-high” concentration feature for the first time in 2020. Therefore, Quanzhou is expected to become a core area that drives the healthy and coordinated development of the surrounding areas with dual efficiency. Ningde exhibited significant “high-high” concentration characteristics in 2014, but did not show any significant spatial effect in other periods. On the other hand, Fuzhou’s coupling coordination degree of dual efficiency did not show significant spatial correlation with the surrounding areas. Overall, the coupling and coordination degree of the dual efficiency in various cities in Fujian Province presents a significant polarization feature, with the overall level of northwest Fujian being low, but the spatial difference is small. Meanwhile, the overall level of southeast Fujian is high, but the spatial difference is significant, and after a decade of development, the spatial distribution pattern has not changed much.

### 3.4. Analysis of the interaction effect between new urbanization efficiency and eco-efficiency based on spatial panel Durbin model (SDM)

The spatial agglomeration and spillover effects of new urbanization efficiency and eco-efficiency are depicted in Fig 6. To further investigate the interaction mechanism of dual efficiency, a spatial panel Durbin model (SDM) with time-fixed effect is employed. The model is defined as follows:

$$\left\{\begin{array}{l} \;\text{ln}\;eco_{it}=\rho \sum_{j=1}^{n}w_{ij}\;\text{ln}\;eco_{jt}+\alpha_{1}\;\text{ln}\;city_{it}+\alpha_{2}\sum_{j=1}^{n}w_{ij}\;\text{ln}\;city_{jt}+\sum_{k=1}^{K}\varphi_{k}\;\text{ln}\;x_{kit}+\lambda_{t}+\varepsilon_{it}\\ \;\text{ln}\;city_{it}=\delta \sum_{j=1}^{n}w_{ij}\;\text{ln}\;city_{jt}+\beta_{1}\;\text{ln}\;eco_{it}+\beta_{2}\sum_{j=1}^{n}w_{ij}\;\text{ln}\;eco_{jt}+\sum_{k=1}^{K}\gamma_{k}\;\text{ln}\;x_{kit}+\xi_{t}+\mu_{it} \end{array}\right.$$ (6)

Where *w*_*ij*_ is the inverse distance space weight matrix, *λ*_*t*_ and *ξ*_*t*_ denote time effects, *ε*_*t*_ and *μ*_*t*_ denote random disturbance terms. *eco* represents eco-efficiency, *city* represents new urbanization efficiency, $\sum_{k=1}^{K}x_{k}$ denotes the *K* control variables. *ρ* and *δ* denote the spatial autoregressive coefficients, which responds to the influence of the dependent variable of the surrounding neighboring areas on the dependent variable of this area. *α*_2_ and *β*_2_ denote the coefficient of the spatial lag term, which responds to the influence of the independent variables in the neighboring areas on the dependent variable of this area. Other symbols represent regression coefficients of explanatory and control variables. Four control variables are chosen to capture the impact of science and technology investment: the degree of openness, industrial structure upgrading, and government intervention. The proportion of science and technology allocation to fiscal expenditure (SCI) measures science and technology investment, while the proportion of foreign direct investment to GDP (FDI) captures the degree of openness. The industrial structure upgrading coefficient (ISU), as proposed by Wang *et al*. [29], is used to represent industrial structure upgrading. The proportion of general public budget expenditure to GDP (GOV) serves as a proxy for government intervention. The data used in the analysis are sourced from the Statistical Yearbook of China’s Cities, the Statistical Yearbook of Fujian Province, and all cities. To avoid heteroscedasticity, all data are logged. Overall, the SDM with a time-fixed effect can provide insights into the relationship between eco-efficiency and new urbanization efficiency, as well as the impact of various control variables on dual efficiency in Fujian Province.

Stata 16.0 is used to build the model, and the results are reported in Table 6. In Table 6, model (1)’s explained variable is new urbanization efficiency, while model (2)’s explained variable is eco-efficiency. The findings show a positive feedback loop between eco-efficiency and new urbanization efficiency in the same location. Local new urbanization efficiency will increase by 0.5988% for every 1% increase in eco-efficiency. The local eco-efficiency will instead rise by 0.1286% for every 1% increase in new urbanization efficiency. Additionally, the univariate spatial autocorrelation test’s conclusion that the increase in new urbanization efficiency and eco-efficiency in the surrounding areas will respectively drive the urbanization and eco-efficiency is supported by the spatial spillover effect of the new urbanization efficiency and eco-efficiency, which is positive (0.3610 and 0.2368), the former being significant at the level of 1% and the latter being significant at the level of 10%. In the new urbanization efficiency Eq (1), the eco-efficiency has a positive spatial spillover effect, which means that for every 1% increase in the eco-efficiency of the surrounding areas, the new urbanization efficiency of the region will increase by 0.3448%. However, this effect has not passed the 5% significance test, indicating that it is not immediately apparent. The new urbanization efficiency has a considerable positive geographic spillover effect on the eco-efficiency, as shown in Eq (2) for eco-efficiency. The eco-efficiency of the area will grow by 0.4271% for every 1% increase in the new urbanization efficiency in the surrounding areas. From the standpoint of the control factors, technological investment, the degree of globalization, governmental intervention, and industrial structure upgrading can favorably influence the new urbanization efficiency, although the function of industrial structure upgrading is not important. There are other aspects that have a substantial positive impact on eco-efficiency in addition to the region’s degree of openness to the outside world.

**Table 6:** Model estimation results.

Variable	Model (1)	Model (2)
lncity		0.1286**[0.0509]
lneco	0.5988***[0.2107]	
lnSCI	0.0485*[0.0401]	0.0699***[0.0182]
lnFDI	0.0032[0.0222]	-0.0018[0.0070]
lnISU	1.1004**[0.4472]	0.7472***[0.2076]
lnGOV	0.2596***[0.0688]	0.0684*[0.0377]
W*lncity	0.3610***[0.2221]	0.4271***[0.1606]
W*lneco	0.3448[0.6650]	0.2368*[0.2151]
W*lnSCI	0.2902**[0.1345]	-0.01096*[0.0661]
W*lnFDI	-0.0266[0.0659]	0.0933***[0.0321]
W*lnISU	-1.1465[1.1348]	-0.7115 [0.5846]
W*lnGOV	-0.0004[0.0028]	0.1996[0.1543]
R^2^	0.3194	0.6198
Log-L	93.4984	93.4984

Note: Data in brackets are standard errors for coefficient estimation.

*, **, and *** indicate significant at the significance level of 10%, 5%, and 1%, respectively.

## 4. Discussion

### 4.1. Rational allocation of resources and intensive development are the weaknesses of new urbanization construction in resource-based areas in Fujian Province

The results of the study show that all cities have reached a level of eco-efficiency that is very effective. Among the cities, Xiamen has recorded the highest new urbanization efficiency, followed by Quanzhou and Fuzhou, whereas Longyan and Nanping have recorded the lowest efficiency. These findings align with the conclusions drawn by Su *et al*. [21]. Throughout the study period, only Longyan (in 2010 and 2016) and Nanping (in 2010) have experienced inefficiencies. Based on the computation of input and output index relaxation variables, the primary causes of inefficiency in new urbanization in 2010 were due to heavy investment redundancy, particularly in capital investment, resource investment, and energy investment. Furthermore, the expected output, mainly population urbanization, economic urbanization, and social urbanization, were insufficient, while the unexpected output, mainly carbon emissions, were excessive. In 2016, the problem of input redundancy remained severe, especially with the serious excess of resources and energy input. However, the issue of unexpected output excess has slightly improved. This indicates that the allocation of resources and energy elements during the new urbanization construction in Longyan and Nanping was ineffective and unreasonable, resulting in low efficiency. To ensure the smooth realization of the high-quality development goal of resource-based areas in Fujian Province, the regions of Longyan and Nanping need to enhance and optimize the utilization of resources. This can be achieved through the scientific and intensive exploitation of resources, improvement of the comprehensive utilization of resources, and maximizing the spatial agglomeration effect of new urbanization efficiency and eco-efficiency. These regions should actively integrate into the construction of Fuzhou Metropolitan Circle and Xiazhangquan Metropolitan Circle and coordinate the development with surrounding cities. During the process of new urbanization construction and green transformation and development, the rationality and effectiveness of input and output should be continually improved.

### 4.2. Resource endowments, location factors and development stages are important factors that affect the new urbanization efficiency and eco-efficiency, as well as the spatial and temporal differences of their coupling coordination development

In the process of promoting urbanization, regional economic development, and industrial structure, resource endowment, geographical location, and development concepts will inevitably lead to regional differences in urbanization efficiency. Coastal areas such as Fuzhou, Xiamen, and Quanzhou have flat terrain, developed transportation, a strong population agglomeration effect, and high levels of economic development, resulting in relatively high urbanization efficiency. These areas have also emphasized green development in industries and strict control of pollution emissions, resulting in high eco-efficiency levels. In contrast, resource-based areas such as Nanping, Longyan, and Sanming have more mountains and hills, underdeveloped transportation, high population emigration, and limited capacity to attract talents, resulting in low new urbanization efficiency. However, these regions are rich in ecological resources and have a high level of forestry industry agglomeration, laying a solid foundation for eco-efficiency, as seen in the rapid improvement of eco-efficiency in Longyan in recent years. Therefore, Northwest Fujian should leverage its strengths, adhere to the development path of ecological towns and green towns, and realize the coupling and coordinated development of new urbanization efficiency and eco-efficiency. Over time, the dual efficiency of each region has σ convergence, and regional differences have been shrinking, indicating that the advantages of each region are gradually highlighted in the process of urbanization. Quanzhou, as the most populous city in Fujian Province, has nine hundred billion industrial clusters and rich commercial resources. It has continuously promoted the green upgrading of industries and fostered new drivers of green development, resulting in outstanding new urbanization efficiency and eco-efficiency. The coupling coordination degree of dual efficiency is second only to Xiamen, with a significant “high-high” concentration characteristic, indicating that Quanzhou adheres to the concept of coordinated development in the process of new urbanization and green development. It has also leveraged the regional agglomeration effect, achieved a win-win situation with the development of new urbanization and ecological civilization in Xiamen, and is expected to develop into a core area that drives the healthy and coordinated development of the surrounding areas with dual efficiency. However, Zhangzhou has a significant “low-high” agglomeration characteristic of the coupling coordination degree of dual efficiency, with a development level far lower than that of Xiamen and Quanzhou. Therefore, in future development, it should rely on its good geographical and industrial advantages, and keep pace with the progress of Xiamen and Quanzhou. The new-type urbanization process in Fujian Province has entered the stage of upgrading and shifting, with a spatial pattern of urbanization of “two poles, two belts, three axes, and six bays” gradually forming. While promoting urbanization construction and economic development, all regions should pay attention to fair competition with surrounding areas, achieve the equal exchange of human resources, land, science and technology, and other resource elements, and ensure the reasonable and balanced allocation of public resources to achieve a win-win situation.

### 4.3. Dredge the interaction between the new urbanization efficiency and eco-efficiency, is an effective way to realize the coupling, coordination and sustainable development of the two

Previous studies have suggested that increasing urbanization can put pressure on the ecological environment [16, 17]. However, the SDM constructed in this paper indicates that new urbanization efficiency and eco-efficiency in the same region have a mutually reinforcing relationship. While previous studies have noted that urbanization can stress the ecological environment, the ecological environment can also constrain urbanization’s development. Fang et al.’s research conclusion [30] aligns with this paper’s findings, which suggest that ecological environment problems are not primarily caused by urbanization, but instead hinge on whether urbanization development and ecological environment are coordinated. During the study period, Xiamen’s new urbanization efficiency and eco-efficiency consistently ranked ahead of other regions, belonging to the “high-high” stable DMU with dual efficiency. The improvement of urbanization efficiency did not reduce eco-efficiency but rather showed a trend of mutual promotion between the two. This outcome is inseparable from the new urbanization path and development model that Xiamen has pursued, which relies on the efficient use of resources, including water, land, energy, and means of production. Effective allocation and integration of resources via recycling and eco-friendly practices is a vital way to improve eco-efficiency. Improving eco-efficiency can promote green transformation of the social economy from “end treatment” to “source prevention and control,” thus advancing traditional extensive economic development models towards more intensive, innovative, and efficient ones. In contrast, improving new urbanization efficiency guarantees human resources, capital, technology, and industrial agglomeration to support eco-efficiency, which ultimately increases living standards, fosters spiritual civilization, raises ecological awareness, and improves eco-efficiency. New urbanization efficiency and eco-efficiency have a positive spatial spillover effect on each other, meaning the improvement of new urbanization efficiency (or eco-efficiency) in surrounding areas will boost the eco-efficiency (or new urbanization efficiency) in the region. While improvement in new urbanization efficiency in surrounding areas may divert production factors (such as talent flow or high-tech spillover) to the region, it can ultimately promote the region’s new urbanization efficiency. Likewise, improving eco-efficiency in surrounding areas can strengthen the cooperation and competition with the local government and improve the ecological environment of the region, indirectly promoting the improvement of the level of new urbanization in the region. However, the spatial spillover effect of eco-efficiency on new urbanization efficiency did not pass the statistical significance test of 5% in this study. Additionally, the spatial autocorrelation test results show that new urbanization efficiency and eco-efficiency in Fujian Province have a significant spatial agglomeration effect, with the former being higher than the latter. Therefore, in new urbanization construction, it is necessary to leverage its agglomeration effect, accelerate the transformation of research and development investment into scientific and technological achievements, realize the upgrading of industrial structure, drive the common improvement of regional urbanization efficiency, strengthen the integration and linkage between regions, promote a reasonable and effective pattern of co-construction, sharing, and win-win of resources and ecology, and ultimately achieve coupling, coordination, and sustainable development of new urbanization efficiency and eco-efficiency, leading to common prosperity.

## 5. Conclusions

This study examines the efficiency and eco-efficiency of new urbanization in nine cities in Fujian Province using the unexpected super-efficiency SBM model, and analyzes their spatial and temporal distribution patterns, as well as their coupling and coordinated development and interaction mechanisms. The main findings are as follows:

Firstly, between 2010 and 2020, the new urbanization efficiency and eco-efficiency in Fujian Province exhibited a fluctuating but generally upward trend, with both indicators being effective (averaging over 1) and significant regional differences, with southeast Fujian exhibiting significantly higher efficiency values than northwest Fujian. The dual efficiency of each city displayed dynamic differentiation features and significant spatial correlation. Although the spatial pattern of new urbanization efficiency changed relatively significantly, the dual efficiency among cities is gradually becoming more spatially balanced.

Secondly, the overall coupling degree of dual efficiency in Fujian Province is relatively high, but the level of coordinated development is relatively low. The coupling coordination degree has gradually evolved from near imbalance to barely coordinated. Xiamen has always maintained a high coupling and high-quality coordination of the dual efficiency, while Sanming, Zhangzhou, Nanping, and Longyan exhibit a state of imbalance. There are obvious regional differences in the level of dual efficiency coupling and coordinated development. The high coupling and coordinated areas are mostly concentrated in the southeast of Fujian. In recent years, the gap between the dual efficiency coupling and coordinated development in various regions has gradually narrowed. The coupling coordination degree of dual efficiency shows a strong spatial agglomeration effect, with the northwest of Fujian Province exhibiting significant characteristics of “low-low” agglomeration, and Quanzhou and Xiamen mainly characterized by “high-high” concentration, with Quanzhou gradually developing into a core area driving the coordinated development of new urbanization and ecological environment in the surrounding areas.

Thirdly, there is a positive interaction between new urbanization efficiency and eco-efficiency, and the spatial spillover effects of both are significantly positive (0.3610 and 0.2368), that is, the new urbanization efficiency and eco-efficiency in surrounding areas respectively contribute to the improvement of the new urbanization efficiency and eco-efficiency in the region. In terms of interactive spatial effect, new urbanization efficiency has a significant spatial spillover effect on eco-efficiency (0.4271), while the spatial spillover effect of eco-efficiency on new urbanization efficiency is not significant.

Overall, this study provides insights into the efficiency and eco-efficiency of new urbanization in Fujian Province, and highlights the importance of coupling and coordinated development in driving sustainable urbanization. These findings can contribute to the development of effective urbanization policies and strategies in Fujian Province and beyond.

## Supporting information

S1 Data (ZIP)
